# Exploring early combination strategy in Latin American patients with newly diagnosed type 2 diabetes: a sub-analysis of the VERIFY study

**DOI:** 10.1186/s13098-021-00686-9

**Published:** 2021-06-15

**Authors:** Sérgio Vencio, Juan P. Manosalva, Chantal Mathieu, Pieter Proot, Hernan Yupanqui Lozno, Päivi M. Paldánius

**Affiliations:** 1 Federal University of Goiás—Post Graduation Programme, Goiânia, Brazil; 2 ICF, Pharmaceutical Institute of Science, Av. Rio Verde, S/N - Cidade Vera Cruz, Aparecida de Goiânia, GO 74935-530 Brazil; 3 Novartis Andean Cluster, Bogotá, Colombia; 4 Department of Endocrinology, UZ Gasthuisberg, University of Leuven, Leuven, Belgium; 5 Novartis Pharma AG, Basel, Switzerland; 6 Colombian Obesity Association-FUNCOBES, Bogota, Colombia; 7 Children’s Hospital, Helsinki University and Helsinki University Hospital, Helsinki, Finland; 8 Research Program for Clinical and Molecular Metabolism, University of Helsinki, Helsinki, Finland

**Keywords:** Early combination, Latin America, Type 2 diabetes mellitus, Vildagliptin

## Abstract

**Background:**

Patients with type 2 diabetes mellitus (T2DM) from Latin American countries face challenges in access to healthcare, leading to under-diagnosis, under-achievement of glycemic target, and long-term complications. Early diagnosis and treatment initiation are of paramount importance in this population due to the high prevalence of risk factors such as obesity and metabolic syndrome. The VERIFY study in patients with newly diagnosed T2DM (across 34 countries), assessed the normoglycemic durability (5 years), with early combination (EC) therapy approach versus the traditional stepwise approach of initiating treatment with metformin monotherapy (MET). Here we present the results from the VERIFY study for participants from eight countries in Latin America.

**Methods:**

Newly diagnosed adult patients with T2DM, HbA1c 6.5–7.5% and body-mass index (BMI) of 22–40 kg/m^2^ were enrolled. The primary endpoint was time to initial treatment failure (TF; HbA1c ≥ 7.0% at two consecutive scheduled visits 13 weeks apart). Time to second TF was evaluated when patients in both groups were receiving and failing on the vildagliptin combination. Safety and tolerability were also assessed for both treatment approaches during the study.

**Results:**

A total of 537 eligible patients (female, 58.8%) were randomly assigned to receive either EC (n = 266) or MET (n = 271). EC significantly reduced the relative risk of time to initial TF by 47% versus MET [HR (95% CI) 0.53 (0.4, 0.7) p < 0.0001]. Overall, 46.4% versus 66.3% of patients achieved the primary endpoint in the EC and MET groups, with a median [interquartile range (IQR)] time to TF of 59.8 (27.5, not evaluable) and 33.4 (12.2, 60.1) months, respectively. The risk for time to second TF was 31% lower with EC (p < 0.0092). A higher proportion of patients receiving EC maintained durable HbA1c < 7.0%, < 6.5%, and < 6.0%. Both treatment approaches were well tolerated, and only 3.2% of participants discontinued the study due to adverse events. All hypoglycemic events (EC: n = 7 and MET: n = 3) were single, mild episodes and did not lead to study discontinuation.

**Conclusion:**

Similar to the global population, long-term clinical benefits were achieved more frequently and without tolerability issues with EC versus standard-of-care MET in this Latin American sub-population.

This study is registered with ClinicalTrials.gov, NCT01528254.

**Supplementary Information:**

The online version contains supplementary material available at 10.1186/s13098-021-00686-9.

## Background

Diabetes presents a major health crisis in Latin American countries, being one of the leading causes of death from a chronic non-communicable disease [1]. In 2019 an estimated 32 million adults from Latin America had diabetes, and the prevalence is projected to increase by 55% in the next 25 years [2]. The disproportionate increase of diabetes in Latin America compared with other Western countries can be attributed to the genetic, socioeconomic, and environmental predisposition of this regional population for various risk factors of type 2 diabetes mellitus (T2DM), such as obesity, insulin resistance and other metabolic disorders such as elevated fasting plasma glucose, impaired glucose tolerance, dyslipidemia and low high-density lipoprotein cholesterol levels [3]. These risk factors are predominant in the Latin American population, with 50% of adults being obese and one-third of the population having metabolic syndrome [4].

Diabetes imposes a high economic burden in Latin American countries, incurring a total cost of USD 70 billion annually and constituting up to 6–24% of the annual total expenditure of the national health budgets [2]. The heterogeneity in the economic vulnerabilities towards diabetes across the region [5] might be due to inequalities in the socioeconomic aspects among countries in the Latin American region [6]. Socioeconomic conditions in Latin America present several public health challenges for diabetes care, such as low disease awareness; inadequate diagnosis; treatment, and preventive measures; and limited access to health care facilities [5, 7, 8]. Diagnosis is often delayed as the prevalence of undiagnosed patients ranges from 10.3 to 50% in this region [9]. Access to treatment is also a major challenge [10], and less than 50% of patients receiving treatment achieve their glycemic targets [11]. In addition, diabetes-related complications predominate in more than 80% of patients with T2DM in this region [11].

Achieving glycemic targets early in the disease continuum leads to a legacy effect of sustained reduction in the risk of complications such as myocardial infarction, death due to any cause, and microvascular disease [12]. A meta-analysis of studies comparing early intensification using combination therapy versus monotherapy showed better glycemic control with early combination therapy [13]. To date, the most compelling evidence signifying the long-term benefits of early intervention with a combination treatment over a 5-year period is available from the VERIFY study [14]. The VERIFY study aimed to determine whether an early combination strategy (building on the molecules with well-established safety and efficacy profiles such as vildagliptin and metformin [15–17]) could provide more durable glycemic and clinical benefits compared with sequentially intensified initial metformin monotherapy in treatment-naïve patients with newly diagnosed T2DM and mild hypoglycemia (a glycated hemoglobin [HbA1c] level of 6.5%–7.5%). Here, we present a sub-analysis of the VERIFY study in patients with T2DM from eight Latin American countries.

## Subjects and methods

### Study design

VERIFY was a phase IV, randomized, double-blind, multi-ethnic, two-arm parallel-group study including treatment-naïve patients with T2DM. Details regarding the study design have been previously published [18].

Briefly, after screening, the eligible patients entered a 3-week “run-in period” during which metformin was individually initiated and/or up-titrated. Patients who were able to tolerate a metformin dose of 1000 mg or higher were randomized 1:1 to receive either early combination (vildagliptin 50 mg plus metformin up to 1000 mg twice-daily) therapy or metformin monotherapy (up to 1000 mg with placebo twice daily) in period 1. Patients with initial loss of glycemic control (determined by two consecutive measurements of HbA1c ≥ 7.0% after randomization, 13 weeks apart), while receiving metformin monotherapy, received vildagliptin as add-on treatment during period 2. Those randomized to the early combination therapy continued to receive the same treatment. Further therapy intensification with open-label insulin in addition to the combination therapy was allowed after treatment failure in period 2, following local diabetes treatment guidelines and at the physician’s discretion (period 3). Patients receiving therapy intensification with blood glucose-lowering drugs, other than insulin in period 3, were discontinued from the study.

### Participants

Adult patients with centrally confirmed HbA1c of 6.5–7.5% and body mass index (BMI) of 22–40 kg/m^2^ were enrolled by 53 centers in eight Latin American countries (Argentina, Brazil, Colombia, Dominican Republic, Guatemala, Mexico, Panama, and Peru).

Patients receiving glucose-lowering treatment (except metformin ≤ 2000 mg daily within one month prior to screening) or using weight-loss medications within three months prior to screening were excluded. Additionally, individuals with contraindications for use of either of the study medications, such as those with chronic liver disease or ongoing congestive heart failure (New York Heart Association Functional Classification III–IV) and those with pregnancy or lactation in progress, were excluded.

### Outcomes

The primary efficacy endpoint was the time to confirmed initial treatment failure, defined as HbA1c ≥ 7.0% at two consecutive planned visits, 13 weeks apart from randomization (the earliest possible failure time is 6 months post-randomization) [14]. Initial treatment failure was compared between the patients receiving early combination (vildagliptin plus metformin) and metformin monotherapy.

The initial treatment failure was also compared between the two treatment groups across the various baseline subgroups including age, gender, race, HbA1c level, BMI and smoking status.

The secondary endpoints included time to second treatment failure, defined as two consecutive values of HbA1c ≥ 7.0% when all patients were receiving combination therapy; change in HbA1c over time, safety and tolerability.

Glycemic control with the two treatment approaches was evaluated over the study duration comparing proportion of patients with HbA1c below 7.0, 6.5 and 6.0%. Changes in body weight throughout the study period was also compared between the two treatment groups.

Treatment-emergent adverse events (AEs) and serious adverse events (SAEs), including pregnancies and medically significant changes in biochemical and other laboratory parameters, were recorded per initial treatment approach throughout the study along with their severity and relationship to blinded study drug. Hypoglycemic events were separately reported. All patients were provided with a home blood glucose monitor. At the first visit, patients were trained to monitor their blood glucose as well as detect and report hypoglycemia. Apart from typical symptoms of hypoglycemia, a capillary whole blood glucose level of < 50 mg/dL (< 2.8 mM), corresponding to a plasma glucose level of < 56 mg/dL (< 3.1 mM), was considered as a hypoglycemic event. Confirmed hypoglycemic events were also classified based on their severity (G1/G2); i.e., those necessitating assistance were considered to be of Grade 2 severity.

### Statistical analysis

Detailed pre-defined statistical analysis plan was published prior to the primary analysis and unblinding [19]. The time to initial and second treatment failures were measured with Cox proportional hazards regression model which included treatment approach and geographical region as factors and baseline HbA1c as a covariate. Subgroup analyses of the initial treatment failure were done using a Cox regression analyses. Cumulative probabilities of initial and second treatment failures over time were assessed using Kaplan–Meier estimates. Patients receiving at least one randomized dose of either study drug or with at least one post-randomization visit for glycemic efficacy contributed to the Kaplan–Meier comparator in each group. Safety outcomes were analyzed for all patients who received at least one dose of randomized study medication (vildagliptin or placebo). AEs were summarized as number and proportions of patients having any AE by treatment group and classified by each primary system organ class. A p value of 0.05 (2-sided) was considered significant. The statistical program used was SAS (versions 9.2 and 9.4; Cary, NC, USA).

## Results

### Baseline characteristics

**Fig. 1:**
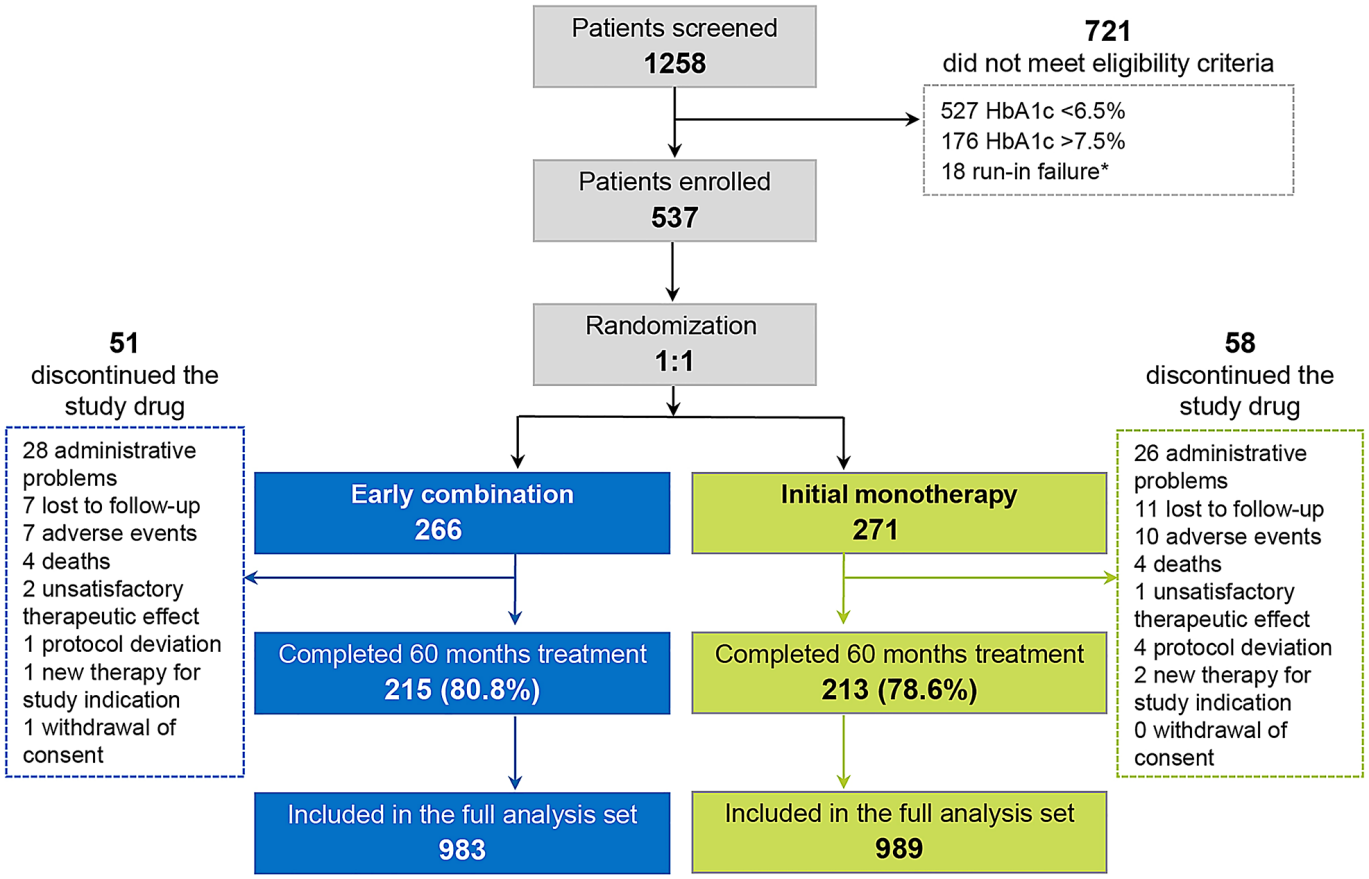
Patients disposition. *HbA1c* glycated haemoglobin. * intolerant to at least 1000 mg daily metformin or not compliant

The VERIFY global population included 2001 randomized patients [14], 537 of whom were from the Latin American region. Of 1258 patients screened from this region, 55.8% (n = 703) failed the screening due to HbA1c value outside of the indicated glycemic range (HbA1c < 6.5% or > 7.5%); 176 (13.9%) had HbA1c > 7.5% with an average HbA1c of 8.9%, ranging from 7.7 to 15.0% (Fig. 1).

A total of 266 patients received early combination (vildagliptin plus metformin), and 271 patients received initial monotherapy. Overall, 80.8% (n = 215) of patients in the early combination and 78.6% (n = 213) of patients in initial monotherapy group completed the study. Approximately 20.3% (n = 109) of the study population discontinued the study prematurely, mostly for administrative reasons (n = 54) such as moving to a new city/country for work or studies or general work-related burden/ unable to attend further visits.

The mean (SD) age of the Latin American study population was 52.7 (9.9) years, with a mean (SD) BMI of 31.0 (4.7) kg/m^2^. The absolute weight of the patients ranged between 44.8 and 142.4 kg. Of the 537 study participants, 316 (58.8%) were females. One in 10 patients (10.2%, n = 55) was diagnosed with T2DM before the age of 40 years. The majority (80.8%, n = 434) of patients included were aged between 40 and 65 years at study enrollment. Among the 537 patients randomized, Native American (39%) or Caucasian (35%) participants were predominant. A total of 27.9% (n = 150) of the population had HbA1c ≥ 7.0% at baseline, and patients had a median [inerquartile range (IQR)] duration of diabetes of 4.3 (1.3, 11.5) months (Table 1).

**Table 1:** Patient characteristics and disposition

Baseline characteristics	Latin American region			Global
	Early combination (n = 266)	Initial monotherapy (n = 271)	Total (N = 537)	Total (N = 2001)
Age (years), mean (SD)	52.0 (10.0)	53.4 (9.7)	52.7 (9.9)	54.3 (9.4)
Age group, n (%)				
< 40	31 (11.7)	24 (8.9)	55 (10.2)	159 (8.0)
40 − < 65	214 (80.5)	220 (81.2)	434 (80.8)	223 (11.1)
≥ 65	21 (7.9)	27 (10.0)	48 (8.9)	1619 (80.9)
Women, n (%)	153 (57.5)	163 (60.1)	316 (58.8)	1060 (53.0)
Predominant race, n (%)				
Native American	102 (38.3)	107 (39.5)	209 (38.9)	210 (10.5)
Caucasian	96 (36.1)	91 (33.6)	187 (34.8)	1217 (60.8)
Median (IQR) duration of T2DM (months)	4.2 (1.6, 11.1)	4.4 (1.1, 11.9)	4.3 (1.3, 11.5)	3.3 (0.9, 10.0)
HbA1c (%)^a^				
Mean (SD)	6.7 (0.5)	6.7 (0.5)	6.7 (0.5)	6.7 (0.5)
˂7.0%, n (%)	191 (71.8)	196 (72.3)	387 (72.1)	1427 (71.3)
≥ 7.0%, n (%)	75 (28.2)	75 (27.7)	150 (27.9)	572 (28.6)
Median (IQR) FPG (mmol/L)^a^	6.6 (5.8, 7.4)	6.6 (5.8, 7.5)	6.6 (5.8, 7.5)	6.9 (6.1, 7.8)
BMI (kg/m^2^)				
Mean (SD)	31.0 (4.6)	31.0 (4.7)	31.0 (4.7)	31.1 (4.7)
< 30 kg/m^2^, n (%)	122 (45.9)	123 (45.4)	245 (45.6)	875 (43.7)
≥ 30 kg/m^2^, n (%)	144 (54.1)	148 (54.6)	292 (54.4)	1126 (56.3)
GFR (mL/min/1.73 m^2^)^b^				
Normal (˃80)	202 (75.9)	217 (80.1)	419 (78.0)	1321 (66.1)
Mild (≥ 50– ≤ 80)	64 (24.1)	54 (19.9)	118 (22.0)	670 (33.5)
Median (IQR) weight (kg)	77.8 (69.0, 92.5)	78.4 (68.0, 90.0)	78.0 (68.5, 91.5)	84.3 (72.3, 97.0)
Current smokers, n (%)	32 (12.0)	29 (10.7)	61 (11.4)	290 (14.5)
Discontinued study participation prematurely, n (%)	51 (19.2)	58 (21.4)	109 (20.3)	403 (20.1)

Baseline refers to randomization visit

*HbA1c* glycated hemoglobin, *BMI* body mass index, *FPG* fasting plasma glucose, *GFR* glomerular filtration rate, *IQR* interquartile range, *SD* standard deviation, *T2DM* type 2 diabetes mellitus

^a^Baseline values were obtained on screening (day 1) or at a later visit (scheduled or unscheduled) if the Day 1 measurements were missing. Two patients in the early combination therapy group did not have baseline HbA1c and fasting plasma glucose measurements on or prior to randomization

^b^Baseline GFR was calculated using the Modification of Diet in Renal Disease Study equation. Serum creatinine and body weight measurements were obtained on Day 1 or at a later visit (scheduled or unscheduled) if the Day 1 measurements were missing

### Outcomes

**Fig. 2:**
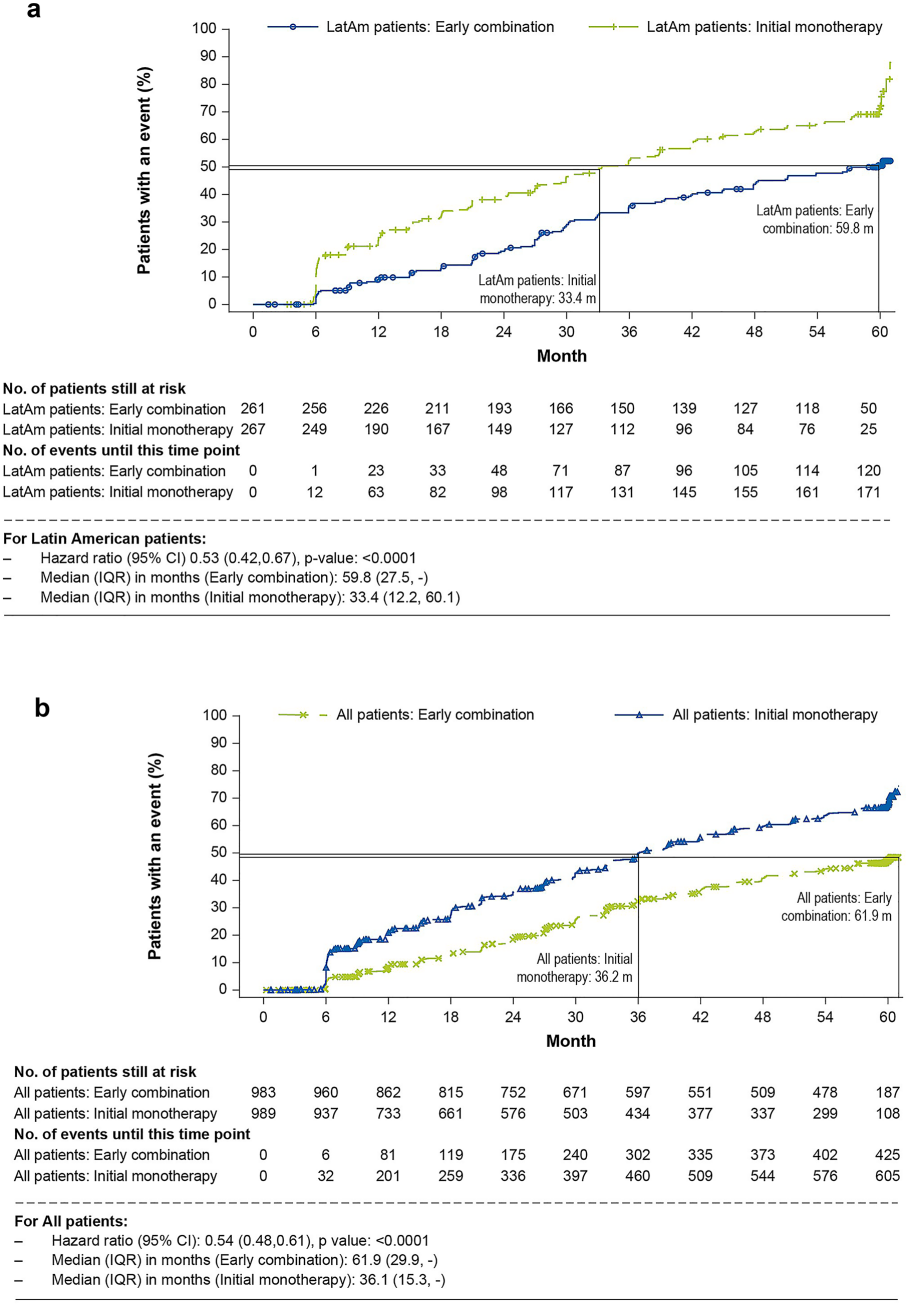
**a** Primary treatment failure* among Latin American patients randomized to early combination versus initial monotherapy. *CI* confidence interval, *HR* hazard ratio, *LatAm* Latin America. *Primary treatment failure is defined as HbA1c ≥ 7.0% at two consecutive scheduled visits, starting from 13 weeks after randomization. The time to initial treatment failure is the time from randomization to the second consecutive scheduled visits with HbA1c ≥ 7.0%. Patients who discontinued the study for any reason during period 1 were censored at the date of discontinuation. Patients with HbA1c < 7.0% (or whose measurement ≥ 7.0% was not confirmed at next scheduled visit) were censored at the date of last study visit. The Kaplan–Meier estimates were performed for patients who had received at least one randomized medication and one post-randomization efficacy parameter assessed. **b** Primary treatment failure* among all patients from VERIFY randomized to early combination versus initial monotherapy. CI, confidence interval; HR, hazard ratio. *Primary treatment failure is defined as HbA1c ≥ 7.0% at two consecutive scheduled visits, starting from 13 weeks after randomization. The time to initial treatment failure is the time from randomization to the second consecutive scheduled visits with HbA1c ≥ 7.0%. Patients who discontinued the study for any reason during period 1 were censored at the date of discontinuation. Patients with HbA1c < 7.0% (or whose measurement ≥ 7.0% was not confirmed at next scheduled visit) were censored at the date of last study visit. The Kaplan–Meier estimates were performed for patients who had received at least one randomized medication and one post-randomization efficacy parameter assessed

At the end of 5 years, 46.4% (n = 121) of patients in the early combination group had met the criteria for initial treatment failure compared with 66.3% (n = 177) in the monotherapy group. Similar to the global results, the median (IQR) time to initial treatment failure was 59.8 (27.5, not evaluable) months in the early combination group compared with 33.4 (12.2, 60.1) months in the monotherapy group. A significant reduction in the risk for time to initial treatment failure was observed in the early combination group compared with monotherapy group over the 5-year study duration [hazard ratio (HR) 0.53 [95% confidence interval (CI): 0.42–0.67); p < 0.0001] (Fig. 2a, b).

**Fig. 3:**
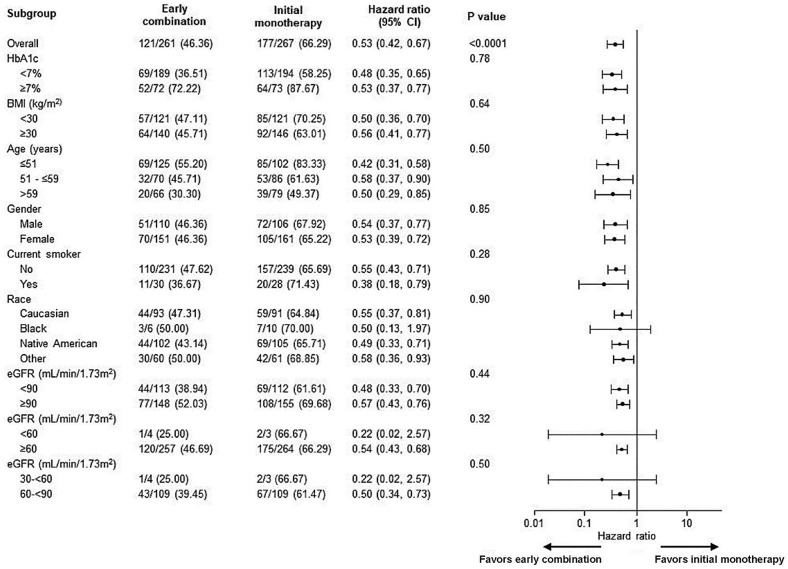
Subgroup analysis of time to initial treatment failure. *BMI* body mass index, *CI* confidence interval, *eGFR* estimated glomerular filtration rate, *HR* hazard ratio. HRs and the associated CIs and p values were obtained from a Cox proportional hazards model containing terms for treatment approach, geographical region, and baseline HbA1c. Significance was established on the basis of a two-sided 0.05 significance level. The treatment-by-subgroup interaction p values are provided for tests of homogeneity of between-group differences among subgroups, with no adjustment for multiple testing. The p value for treatment comparison in the overall population is also provided. *BMI* body mass index, *CI* confidence interval, *GFR* glomerular filtration rate, *HbA1c* glycated hemoglobin, *HR* hazard ratio, *N* total number of patients considered for each subgroup analysis, *n* number of patients with relevant results within each subgroup

The subgroup analysis for time to initial treatment failure for all pre-defined baseline variables, such as age, HbA1c at screening or race, with both treatment approaches is presented in Fig. 3. Across all pre-defined subgroups, the risk for initial treatment failure was lower with early combination therapy.

**Fig. 4:**
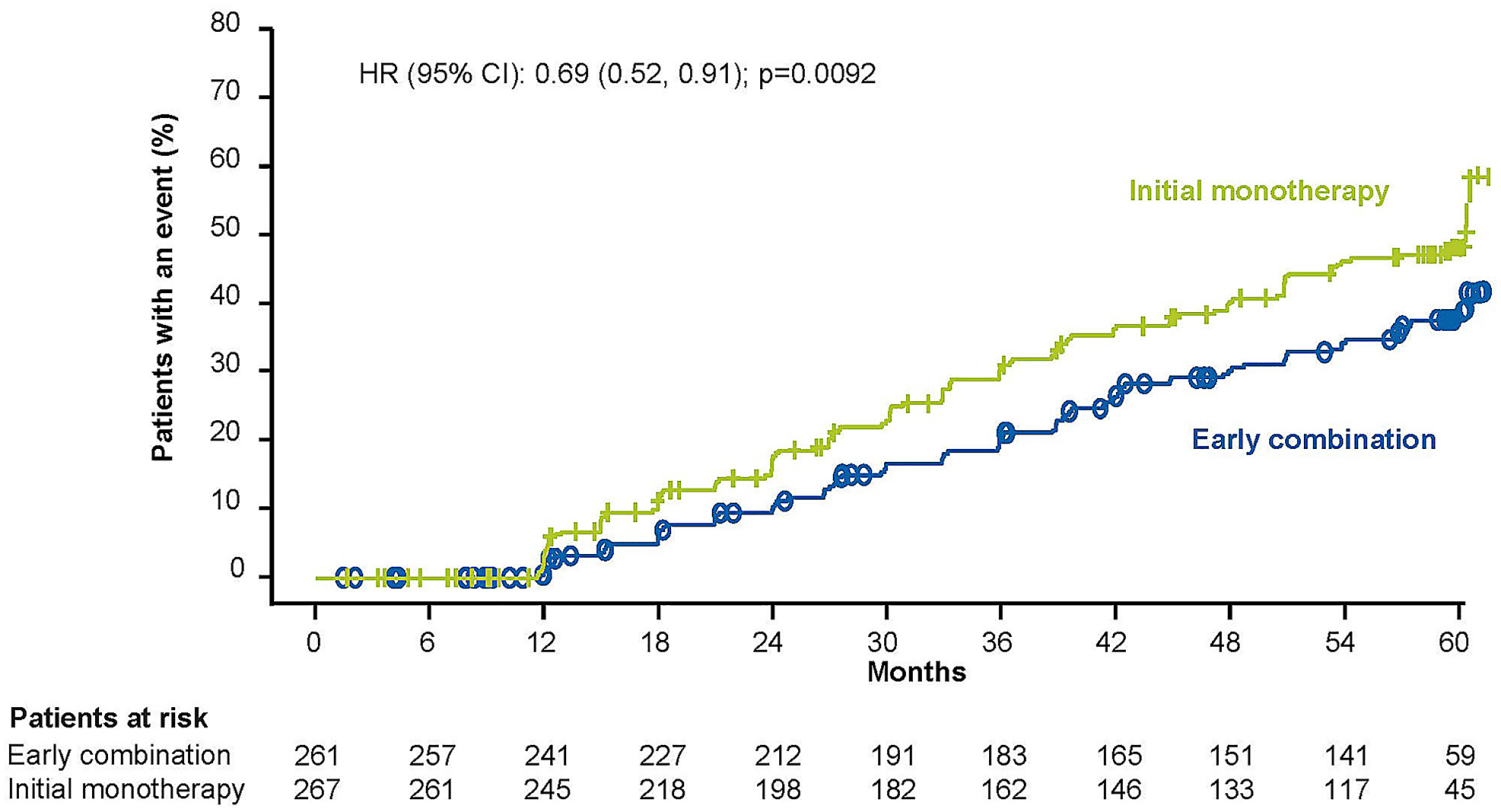
Secondary treatment failure* among patients with early combination versus initial monotherapy followed by vildagliptin addition. *CI* confidence interval, *HbA1c* glycated hemoglobin; HR, hazard ratio. *Secondary treatment failure is defined as two consecutive scheduled visits with HbA1c ≥ 7.0% during period 2 (i.e., after period 1 comparing metformin monotherapy versus early combination therapy with metformin and vildagliptin and up to end of period 2 when both groups are on combination therapy after primary treatment failure. The time to secondary treatment failure is the number of days from randomization to the second confirmed HbA1c ≥ 7.0% during consecutive scheduled visits, three months apart, in period 2. The Kaplan Meier estimates were performed for patients who had received at least one randomized medication and one post-randomization efficacy parameter assessed. Patients who had no event and discontinued the study for any reason during period 1 or period 2 were censored at the date of discontinuation. Patients who entered period 3 from period 1 were censored to last study visit prior to start of period 3. Two-sided p value was obtained from a Cox proportional hazards model containing terms for treatment approach. Baseline HbA1c was the value obtained on Day 1, or the value obtained at an earlier visit (scheduled or unscheduled) which was closest to Day 1, if Day 1 measurement was missing

**Fig. 5:**
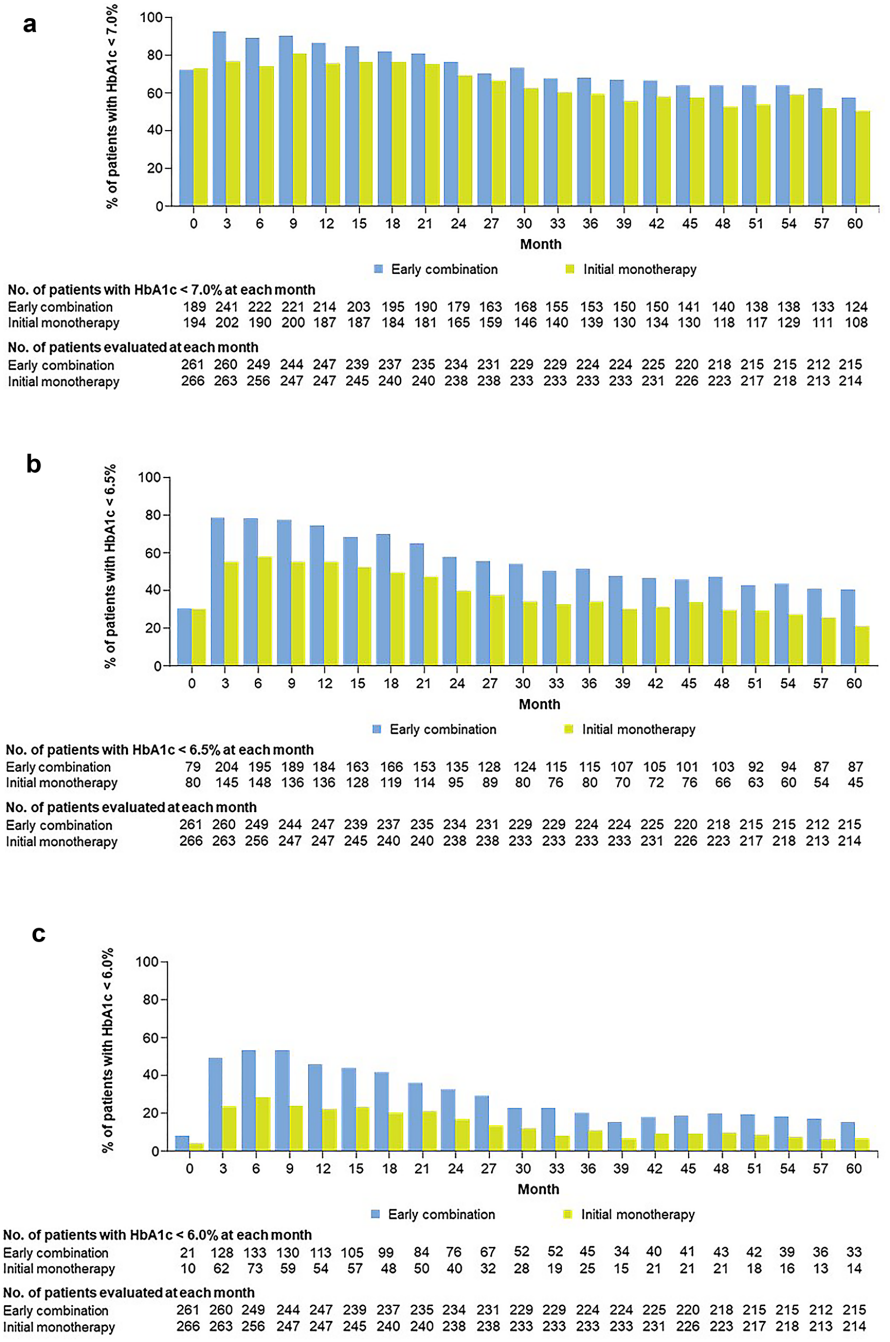
**a** Success rate of early combination and initial monotherapy approaches at cut-off HbA1c 7.0%. *HbA1c* glycated hemoglobin. **b** Success rate of early combination and initial monotherapy approaches at cut-off HbA1c 6.5%. HbA1c, glycated hemoglobin. **c** Success rate of early combination and initial monotherapy approaches at cut-off HbA1c 6.0%. *HbA1c* glycated hemoglobin

During period 2, 33.7% (n = 88) of patients in the early combination group experienced a secondary treatment failure, versus 43.4% (n = 116) of patients in the monotherapy group. A significant reduction in risk of secondary treatment failure was observed with the early combination group compared with the monotherapy group [HR (95% CI) 0.69 (0.52, 0.91); p = 0.0092] (Fig. 4). Additionally, a higher proportion of patients in the early combination group compared with the initial monotherapy group had HbA1c values < 7.0%, < 6.5% and < 6.0% over five years (Fig. 5a–c).

Across the study period, body weight remained stable in both treatment groups. The median (IQR) body weight at baseline was 77.6 (68.3–91.0) kg and at the end of study was 77.0 (67.9–89.9) kg (Additional file 1: Figure S1).

The overall safety and tolerability profiles were similar between treatment approaches, with no unexpected safety findings reported. The incidence of AEs and SAEs over five years among Latin American participants between the two treatment approaches were 240 (89.8%) and 37 (13.8%), respectively, in the combination treatment group and 230 (85.2%) and 41 (15.2%), respectively, in the initial monotherapy group. Most of the AEs were of mild nature and a small fraction of AEs was reported to be related to the study treatment (< 4%). Only 2.6% (n = 7) of patients in early combination and 3.7% (n = 10) of patients in initial monotherapy group discontinued the treatment due to AEs. Majority of the drug-related AEs were gastrointestinal disorders likely to be related to metformin. Four patients in each group died during the study, but no deaths were considered to be related to any of the study drugs (Table 2).

**Table 2:** Adverse events by preferred terms in the Latin American study population

Patients with AEs, n (%)	Early combination n = 267 n (%)	Initial monotherapy n = 270 n (%)
Patients with at least one AE	240 (89.8)	230 (85.2)
Diarrhea	47 (17.6)	36 (13.3)
Back pain	41 (15.4)	32 (11.9)
Influenza	41 (15.4)	24 (8.9)
Arthralgia	40 (15.0)	43 (15.9)
Urinary tract infection	34 (12.7)	29 (10.7)
Hypertension	34 (12.7)	39 (14.4)
Nasopharyngitis	33 (12.4)	38 (14.1)
Headache	31 (11.6)	28 (10.4)
Pain in extremity	31 (11.6)	42 (15.6)
Anxiety	18 (6.7)	31 (11.5)
Dyslipidemia	23 (8.6)	27 (10.0)
Pharyngitis	21 (7.9)	22 (8.1)
Hypertriglyceridemia	20 (7.5)	10 (3.7)
Bronchitis	16 (6.0)	13 (4.8)
Gastroenteritis	14 (5.2)	16 (5.9)
Abdominal pain	13 (4.9)	14 (5.2)
Hepatic steatosis	13 (4.9)	9 (3.3)

Patients with multiple adverse events under one treatment approach were counted only once in the adverse event category for that treatment approach

*AE* adverse events

Hypoglycemic events were reported in seven patients in the early combination group and three in the initial monotherapy group, all of which were single events of grade 1 severity and did not lead to study discontinuation. One of the hypoglycemic events in each treatment group occurred during insulin treatment after 33 and 51 months in individuals whose diabetes rapidly progressed. One participant from this Latin American region reported a pregnancy, and consequently, the study treatment (vildagliptin plus metformin) was permanently discontinued; the participant delivered a healthy baby at term.

## Discussion

This regional analysis of the VERIFY study in Latin American participants demonstrated that early combination therapy with vildagliptin plus metformin improved glycemic durability in patients with newly diagnosed T2DM compared with the standard-of-care initial metformin monotherapy followed by sequential combination with vildagliptin. The early combination therapy approach among Latin American participants with a low diagnostic HbA1c (6.5%–7.5%) significantly reduced the risk of initial treatment failure by 47% compared with metformin monotherapy throughout the 5-year study duration. Early combination approach also reduced the risk of secondary treatment failure by 31% compared with timely and immediate intensification of metformin monotherapy based on the mandate as per the study protocol. In addition, the outcomes of this sub-analysis showed the applicability of the benefits of early combination therapy approach across the sub-groups of patients in the Latin American region. Overall, the findings of this regional analysis are consistent with those of the VERIFY global study, despite the distinct predisposing genetic and environmental characteristics of this high-risk population and inclusion of a relatively large proportion of unique, infrequently studied patients of Native Hispanic American origin[3].

The diverseness of baseline characteristics confirms that the VERIFY study protocol genuinely allowed enrollment of a globally heterogeneous population reflective of currently diagnosed individuals with diabetes. In Latin America, as in other global regions outside Northern Europe [20], a deeper analysis of the glycemic data from the screening phase reverberates the challenges in accurate, early and timely diagnosis of T2DM based on glycemia as reported for Latin America [5, 7]. Therefore, in this region where there is less-structured public healthcare infrastructure, socioeconomic inequality, and limited access to care, it is sensible that recommendations and procedures facilitate early diagnosis and intervention.

The majority of the Latin American sub-population from the VERIFY study were relatively young at the time of T2DM diagnosis, between the age of 40 and 65 years, which corresponds to the age range that has been reported to have the sharpest rise in diabetes prevalence in many Latin American countries (Argentina, Chile, Uruguay, and Peru) [21]. The overall inclusion of approximately 10% of young-onset diabetes (YOD) patients (diagnosed before the age of 40 years) in Latin America was similar to the overall VERIFY global study population, whereas the highest proportion of YOD was enrolled from Asia [22]. Although the VERIFY study protocol had limited, if any gender-specific exclusion criteria beyond those related to current pregnancy and breast-feeding, the even more pronounced predominance of female participants in this regional sub-population (Latin American: 59% versus global: 53%) suggests increased disease awareness and health-seeking behavior among women compared to men, supporting similar observations from previous real-world studies from the Latin American region [11]. Therefore, the study population from Latin America included in our sub-analysis represented the overall current demographics of those with newly-diagnosed T2DM within the region.

Obesity in Latin American countries is highly prevalent and is influenced by heterogeneous socioeconomic factors such as lifestyle and diet [23], which may have been the underlying reason for the wide range of body weight (with identical mean BMI value) versus the global, observed in this Latin American sub-population of VERIFY. An earlier real-world study reported that the majority of patients with T2DM from Latin America were obese (BMI ≥ 30 kg/m^2^), and only 46.2% of these obese patients achieved glycemic control with sequential treatment intensification [11]. However, in our regional as well as global study populations, the early combination approach showed glycemic benefits in both sub-groups of patients with BMI ≥ 30 kg/m^2^ and < 30 kg/m^2^. In addition, the overall body weight of the study participants remained stable over 5 years, with both the treatment approaches.

Earlier studies with the traditional sequential treatment intensification approach in clinical practice among patients from Latin America have shown that only 25–30% of patients achieved their glycemic target [10, 11]. A recent review of Latin American studies showed that non-attainment of glycemic control is primarily associated with longer duration of disease, a complex regimen, and inadequate access to healthcare and insurance coverage [9]. In this sub-analysis, 53.6% of patients using early combination therapy achieved and sustained glycemic control over a long term.

The current diabetes management guidelines from the Latin American Diabetes Association (ALAD, Asociación Latinoamericana de Diabetes) recommend early combination therapy only in patients with diagnostic HbA1c value of > 8.0% [24]. However, in clinical practice in Latin America, failing monotherapy treatment is intensified using combination therapies only at an average HbA1c of 8.5% [25]. In addition, < 40% of patients receive combination oral antidiabetics within the first 2 years of T2DM diagnosis [10]. In a cross-sectional study from Colombia, of 363 patients with T2DM with a median HbA1c of 6.8%, only 43.8% received combination therapy at their first consultation, and therapeutic inertia was reported in > 50% of the consultations. Interestingly, the risk of therapeutic inertia at follow-up consultations was low with better HbA1c control [26]. In 2017, a survey-based study from Brazil showed that 75% of physicians do not consider combination therapy at treatment initiation and prefer to use it as a second-line therapy [8]. These observations indicate the presence of therapeutic inertia as one of the major barriers to optimized management of glycemia [27].

Based on the clinical benefits of early combination therapy demonstrated by the VERIFY global study, the American Diabetes Association (ADA) and European Association for the Study of Diabetes (EASD) have updated their consensus statement suggesting that healthcare providers should engage in shared decision making around initial combination therapy in new-onset cases of T2DM [28]. Similarly, the latest update of the Brazilian guidelines recommends early combination therapy in treatment-naïve patients with HbA1c 6.5–7.5% to delay treatment failure and improve glycemic control based on the clinical benefits [29].

This Latin American sub-analysis of the VERIFY study has shown that with early combination therapy, patients achieved a durable glycemic control, with a higher proportion maintaining HbA1c levels below 7.0, 6.5, and 6.0% over 5 years compared with those who received initial metformin monotherapy across various sub-groups. These findings suggest that regardless of the disparities in the social situation in Latin America, an early combination strategy can help achieve glycemic control in patients newly diagnosed with T2DM in this region.

### Strengths and limitations

The observed glycemic durability and improved safety parameters confirm the clinical applicability (and generalizability) of the previously presented global results of the benefits of early combination therapy in the Latin American population with newly diagnosed T2DM. These findings strengthen the global clinical applicability of the VERIFY study results. The long-term study duration of 5 years is one of the main strengths of the VERIFY study and its sub-analysis. The pragmatic design of the study made it feasible to include a study population reflective of the current and characteristically diverse T2DM population in the Latin American region, including patients with YOD, obese individuals, and a predominance of female patients who otherwise so often are excluded from clinical studies with no valid reasons [30–32].

## Conclusion

The significant and consistent improvement in the long-term glycemic durability with early combination approach compared with initial metformin monotherapy in patients with newly diagnosed T2DM shown by the VERIFY study was also observed in this Latin American regional analysis. Considering these glycemic benefits, early combination treatment could be an effective approach to address the challenge in attaining the optimal therapeutic target for patients in this region.

## Supplementary Information

**Additional file 1**: **Figure 1**. Body weight of patients in early combination and initial monotherapy groups. BL, baseline. Body weight measures are given in median and interquartile range (vertical bars). Baseline weight is the measurement obtained on Day 1 or on sample obtained on an earlier visit (scheduled or unscheduled). Day is relative to the first day of treatment (Day 1).

## Data Availability

Novartis is committed to sharing with qualified external researchers, access to patient-level data and supporting clinical documents from eligible studies. These requests are reviewed and approved by an independent review panel based on scientific merit. All data provided are anonymized to respect the privacy of patients who had participated in the trial in line with applicable laws and regulations. The criteria and process for trial data availability are described online at https://www.clinicalstudydatarequest.com/

## Abbreviations

ADA: American Diabetes Association
AE: Adverse event
ALAD: Asociación Latinoamericana de Diabetes
BMI: Body mass index
CI: Confidence interval
EASD: European Association for the study of Diabetes
EC: Early combination
HbA1c: Glycated hemoglobin
HR: Hazard ratio
IQR: Interquartile range
MET: Metformin
SAE: Serious adverse event
SD: Standard deviation
TF: Treatment failure
T2DM: Type 2 diabetes mellitus
VERIFY: Vildagliptin Efficacy in combination with metfoRmIn For earlY treatment of Type 2 diabetes

## References

[1] Calazans JA, Queiroz BL (2020). The adult mortality profile by cause of death in 10 Latin American countries (2000–2016). Rev Panam Salud Publica.

[2] International Diabetes Federation. IDF diabetes Atlas ninth edition; 2019. https://www.diabetesatlas.org/en/. Accessed 01 Dec 2020.

[3] Caballero AE (2005). Diabetes in the Hispanic or Latino population: genes, environment, culture, and more. Curr Diab Rep.

[4] Aschner P, Aguilar-Salinas C, Aguirre L, Franco L, Gagliardino JJ, Gorban de Lapertosa S (2014). Diabetes in South and Central America: an update. Diabetes Res Clin Pract.

[5] Gallardo-Rincon H, Cantoral A, Arrieta A, Espinal C, Magnus MH, Palacios C (2020). Review: Type 2 diabetes in Latin America and the Caribbean: Regional and country comparison on prevalence, trends, costs and expanded prevention. Prim Care Diabetes.

[6] Hofman AA (2000). The economic development of Latin America in the twentieth century.

[7] Blasco-Blasco M, Puig-Garcia M, Piay N, Lumbreras B, Hernandez-Aguado I, Parker LA (2020). Barriers and facilitators to successful management of type 2 diabetes mellitus in Latin America and the Caribbean: a systematic review. PLoS ONE.

[8] Vencio S, Paldanius PM, Bluher M, Giannella-Neto D, Caiado-Vencio R, Strain WD (2017). Understanding the barriers and improving care in type 2 diabetes: Brazilian perspective in time to do more in diabetes. Diabetol Metab Syndr.

[9] Aviles-Santa ML, Monroig-Rivera A, Soto-Soto A, Lindberg NM (2020). Current state of diabetes mellitus prevalence, awareness, treatment, and control in Latin America: challenges and innovative solutions to improve health outcomes across the continent. Curr Diab Rep.

[10] Lopez Stewart G, Tambascia M, Rosas Guzman J, Etchegoyen F, Ortega Carrion J, Artemenko S (2007). Control of type 2 diabetes mellitus among general practitioners in private practice in nine countries of Latin America. Rev Panam Salud Publica.

[11] Irazola V, Rubinstein A, Bazzano L, Calandrelli M, Chung-Shiuan C, Elorriaga N (2017). Prevalence, awareness, treatment and control of diabetes and impaired fasting glucose in the Southern Cone of Latin America. PLoS ONE.

[12] Holman RR, Paul SK, Bethel MA, Matthews DR, Neil HA (2008). 10-year follow-up of intensive glucose control in type 2 diabetes. N Engl J Med.

[13] Cai X, Gao X, Yang W, Han X, Ji L (2018). Efficacy and safety of initial combination therapy in treatment-naive type 2 diabetes patients: a systematic review and meta-analysis. Diabetes Ther.

[14] Matthews DR, Paldanius PM, Proot P, Chaing YT, Stumvoll M, Del Prato S (2019). Glycaemic durability of an early combination therapy with vildagliptin and metformin versus sequential metformin monotherapy in newly diagnosed type 2 diabetes (VERIFY): a 5-year, multicentre, randomised, double-blind trial. Lancet.

[15] Diabetes Prevention Program Research Group (2012). Long-term safety, tolerability, and weight loss associated with metformin in the Diabetes Prevention Program Outcomes Study. Diabetes Care.

[16] Mathieu C, Kozlovski P, Paldánius PM, Foley JE, Modgill V, Evans M (2017). Clinical safety and tolerability of vildagliptin—Insights from randomised trials, observational studies and post-marketing surveillance. Eur Endocrinol.

[17] Bosi E, Dotta F, Jia Y, Goodman M (2009). Vildagliptin plus metformin combination therapy provides superior glycaemic control to individual monotherapy in treatment-naive patients with type 2 diabetes mellitus. Diabetes Obes Metab.

[18] Del Prato S, Foley JE, Kothny W, Kozlovski P, Stumvoll M, Paldánius PM (2014). Study to determine the durability of glycaemic control with early treatment with a vildagliptin-metformin combination regimen vs. standard-of-care metformin monotherapy-the VERIFY trial: a randomized double-blind trial. Diabet Med.

[19] Matthews DR, Paldanius PM, Stumvoll M, Han J, Bader G, Chiang YT (2019). A pre-specified statistical analysis plan for the VERIFY study: Vildagliptin efficacy in combination with metformin for early treatment of T2DM. Diabetes Obes Metab.

[20] Chan JC, Vencio S, Proot P, Paldánius PM, Mohan V, et al. Screening values of glycated haemoglobin suggest regional barriers in detecting T2DM early: Analysis of the VERIFY study. Poster presented at Internalthional Diabetes Federation Congress 2019. OP-0279. 10.26226/morressier.5d9b622bea541d6ca8493b20

[21] Shen J, Kondal D, Rubinstein A, Irazola V, Gutierrez L, Miranda JJ (2016). A multiethnic study of pre-diabetes and diabetes in LMIC. Glob Heart.

[22] Chan JC, Paldánius PM, Mathieu C, Stumvoll M, Matthews DR, Del Prato S (2021). Early combination therapy delayed treatment escalation in newly diagnosed young-onset type 2 diabetes: a subanalysis of the VERIFY study. Diabetes Obes Metab.

[23] Filozof C, Gonzalez C, Sereday M, Mazza C, Braguinsky J (2001). Obesity prevalence and trends in Latin-American countries. Obes Rev.

[24] Association of Latin America Diabetes. ALAD guidelines on the diagnosis, control and treatment of type 2 diabetes mellitus with medicine based on evidence. Edition. 2019; https://www.revistaalad.com/guias/5600AX191_guias_alad_2019.pdf. Accessed 01 Dec 2020.

[25] Mendivil CO, Marquez-Rodriguez E, Angel ID*,* Paz G, Rodríguez , Almada J. Comparative effectiveness of vildagliptin in combination with other oral anti-diabetes agents in usual-care conditions: the EDGE-Latin America study. Curr Med Res Opin. 2014; 30: 1769–76. 10.1185/03007995.2014.928274 24867177

[26] Machado-Duque ME, Ramirez-Riveros AC, Machado-Alba JE (2017). Effectiveness and clinical inertia in patients with antidiabetic therapy. Int J Clin Pract.

[27] Guzman JR, Lyra R, Aguilar-Salinas CA, Cavalcanti S, Escaño F, Tambasia M (2010). Treatment of type 2 diabetes in Latin America: a consensus statement by the medical associations of 17 Latin American countries. Latin American Diabetes Association. Rev Panam Salud Publica.

[28] Buse JB, Wexler DJ, Tsapas A, Rossing P, Mingrone G, Mathieu C (2020). 2019 update to: management of hyperglycemia in type 2 diabetes 2018, a consensus report by the American Diabetes Association (ADA) and the European Association for the Study of Diabetes (EASD). Diabetes Care.

[29] Bertoluci MC, Salles JEN, Silva-Nunes J, Pedrosa HC, Moreira RO, Duarte RCDS (2020). Portuguese-Brazilian evidence-based guideline on the management of hyperglycemia in type 2 diabetes mellitus. Diabetol Metab Syndr.

[30] Zucker I, Prendergast BJ (2020). Sex differences in pharmacokinetics predict adverse drug reactions in women. Biol Sex Differ.

[31] Rogers WA, Ballantyne AJ (2008). Exclusion of women from clinical research: myth or reality?. Mayo Clin Proc.

[32] Geller SE, Koch AR, Roesch P, Filut A, Hallgren E, Carnes M (2018). The more things change, the more they stay the same: a study to evaluate compliance with inclusion and assessment of women and minorities in randomized controlled trials. Acad Med.

