# Supplementary figures and images for: Exploring early combination strategy in Latin American patients with newly diagnosed type 2 diabetes: a sub-analysis of the VERIFY study
Source: Diabetol Metab Syndr. 2021 Jun 15;13:68. doi: 10.1186/s13098-021-00686-9 (PMC8207702; doi:10.1186/s13098-021-00686-9)

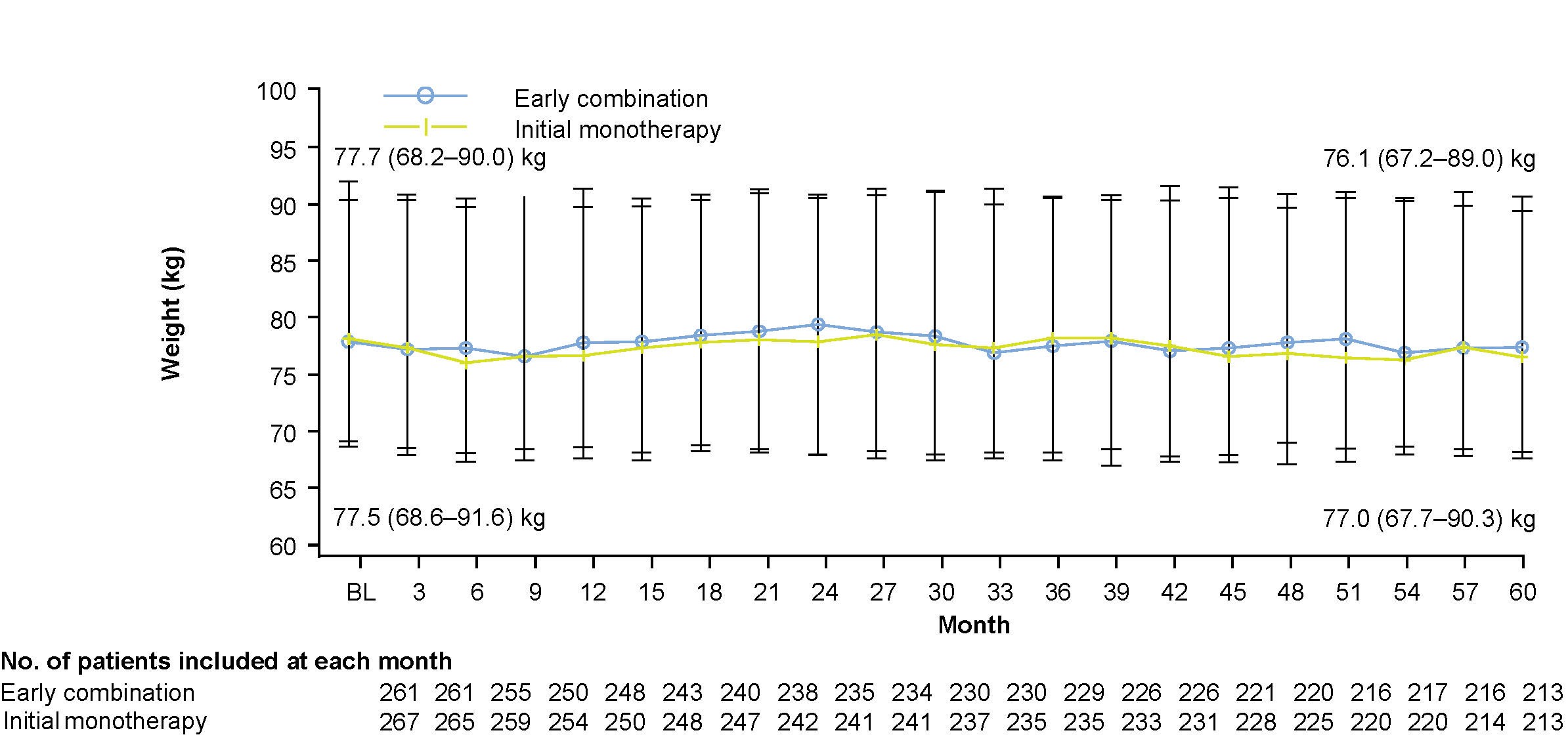

Supplement: Supplementary file 1 — Additional file 1: Figure 1. Body weight of patients in early combination and initial monotherapy groups. BL, baseline. Body weight measures are given in median and interquartile range (vertical bars). Baseline weight is the measurement obtained on Day 1 or on sample obtained on an earlier visit (scheduled or unscheduled). Day is relative to the first day of treatment (Day 1). [file 13098_2021_686_MOESM1_ESM.jpg]
